# Murine embryos exposed to human endometrial MSCs-derived extracellular vesicles exhibit higher VEGF/PDGF AA release, increased blastomere count and hatching rates

**DOI:** 10.1371/journal.pone.0196080

**Published:** 2018-04-23

**Authors:** Rebeca Blázquez, Francisco Miguel Sánchez-Margallo, Verónica Álvarez, Elvira Matilla, Nuria Hernández, Federica Marinaro, María Gómez-Serrano, Inmaculada Jorge, Javier G. Casado, Beatriz Macías-García

**Affiliations:** 1 Stem Cell Therapy Unit, Jesús Usón Minimally Invasive Surgery Centre, Cáceres, Spain; 2 CIBER de Enfermedades Cardiovasculares, Madrid, Spain; 3 Assisted Reproduction Unit, Jesús Usón Minimally Invasive Surgery Centre, Cáceres, Spain; 4 Centro Nacional de Investigaciones Cardiovasculares (CNIC), Madrid, Spain; Academic Medical Centre, University of Amsterdam, NETHERLANDS

## Abstract

Endometrial Mesenchymal Stromal Cells (endMSCs) are multipotent cells with immunomodulatory and pro-regenerative activity which is mainly mediated by a paracrine effect. The exosomes released by MSCs have become a promising therapeutic tool for the treatment of immune-mediated diseases. More specifically, extracellular vesicles derived from endMSCs (EV-endMSCs) have demonstrated a cardioprotective effect through the release of anti-apoptotic and pro-angiogenic factors. Here we hypothesize that EV-endMSCs may be used as a co-adjuvant to improve *in vitro* fertilization outcomes and embryo quality. Firstly, endMSCs and EV-endMSCs were isolated and phenotypically characterized for *in vitro* assays. Then, *in vitro* studies were performed on murine embryos co-cultured with EV-endMSCs at different concentrations. Our results firstly demonstrated a significant increase on the total blastomere count of expanded murine blastocysts. Moreover, EV-endMSCs triggered the release of pro-angiogenic molecules from embryos demonstrating an EV-endMSCs concentration-dependent increase of VEGF and PDGF-AA. The release of VEGF and PDGF-AA by the embryos may indicate that the beneficial effect of EV-endMSCs could be mediating not only an increase in the blastocyst’s total cell number, but also may promote endometrial angiogenesis, vascularization, differentiation and tissue remodeling. In summary, these results could be relevant for assisted reproduction being the first report describing the beneficial effect of human EV-endMSCs on embryo development.

## Introduction

Mesenchymal Stromal Cells (MSCs) are ubiquitous multipotent progenitor cells that can be found in bone marrow, umbilical cord, placenta or adipose tissue among others [1]. Their main features are plastic adherence, high proliferative potential, differentiation potential towards osteogenic, adipogenic and chondrogenic lineages and their self-renewal capacity [2]. Due to their immunomodulatory and anti-inflammatory activities, these cells have been considered for the treatment of a wide variety of clinical conditions including cirrhosis or articular damage [3,4]. However, invasive extraction of MSCs by means of tissue biopsies and the need for later expansion are limiting factors for their clinical application.

MSCs release paracrine factors that have also been shown to effectively mediate tissue repair and regeneration [5] offering a good cell-free alternative to direct MSCs application. Among all the paracrine factors, special attention is being put on exosomes, which are small vesicles (40–150 nm) of endosomal origin that mediate cell to cell communication. These vesicles are known to be composed of RNAs, DNA, lipids and proteins, although these components may vary depending upon cell type and physiological or pathological status [6,7].

Recently, MSCs have been isolated from human menstruation offering the advantage of being a non-invasive source of multipotent cells that can grow twice faster than bone marrow-derived MSCs [1]. This intense proliferative potential is aimed to maintain the dynamic remodeling of the endometrium [8] during the menstrual cycle. This cycle consists of a secretory and a proliferative phase which is followed by a profound desquamation of the endometrium during menstruation, being repeated over 400 times throughout the women’s reproductive life [9]. Hence, endometrial MSCs offer the advantage of being a reliable and cost-effective source of multipotent cells. Recent studies have demonstrated that exosomes derived from menstrual MSCs alleviate apoptosis in a mouse model of fulminant hepatic failure [10] and decrease tumor-induced angiogenesis in prostate PC3 tumor cells [11].

Regarding the role of extracellular vesicles derived from endometrial MSCs (EV-endMSCs) in early pregnancy, it is known that the endometrium establishes a complex interplay with the embryo being this cell to cell communication mediated in part by exosome release [12]. This dynamic communication is partly mediated by cytokines and growth factors that are involved in pregnancy. For example, T cell-derived cytokines such as GM-CSF or IL-3 have been demonstrated to be important growth factors for the trophoblast, while TGF-ß, CSF-1 and LIF are involved in implantation determining embryo survival and viable offspring delivery [13,14].

Preimplantation development requires a transcriptional control for a precise coordination of multiple cell-fate decisions [15]. It requires the reprogramming of parental epigenomes to a totipotent state and the epigenetic programs are essential for lineage decisions and differentiation [16]. Several dynamic changes occurs during blastocyst formation and the polarization model seems to be the best model to incorporate most known information [17]. Once the oocyte is fertilized, the embryo undergoes symmetric and asymmetric divisions during morula to blastocyst transition. When it reaches the expanded blastocyst stage, it will escape from the zona pellucida (embryo hatching), being this a mandatory step for successful implantation [18,19]. Even when endometrial MSCs exosomes are presumed to vehicle many soluble factors including cytokines and growth factors, the effect that these vesicles may exert as coadjutants during embryo culture still remains unexplored. Therefore, the aim of the present study was to isolate and characterize human endometrial MSCs and their extracellular vesicles; then to evaluate their biological effect these EV- MSCs were co-cultured with murine 2 cell embryos produced *in utero*. Embryo development and hatching were *in vitro* evaluated.

Our results firstly demonstrate the beneficial effect of EV-endMSCs on embryo developmental competence, total cell number and embryo hatching. Moreover, the analysis of soluble factors released by co-cultured embryos evidenced a significant increase of VEGF and PDGF-AA secretion which have been associated with enhanced angiogenesis, vascularization, differentiation and tissue remodeling possibly aiming to enhance endometrial receptivity.

## Materials and methods

### Isolation and *in vitro* expansion of endometrial mesenchymal stromal cells

**Fig 1:**
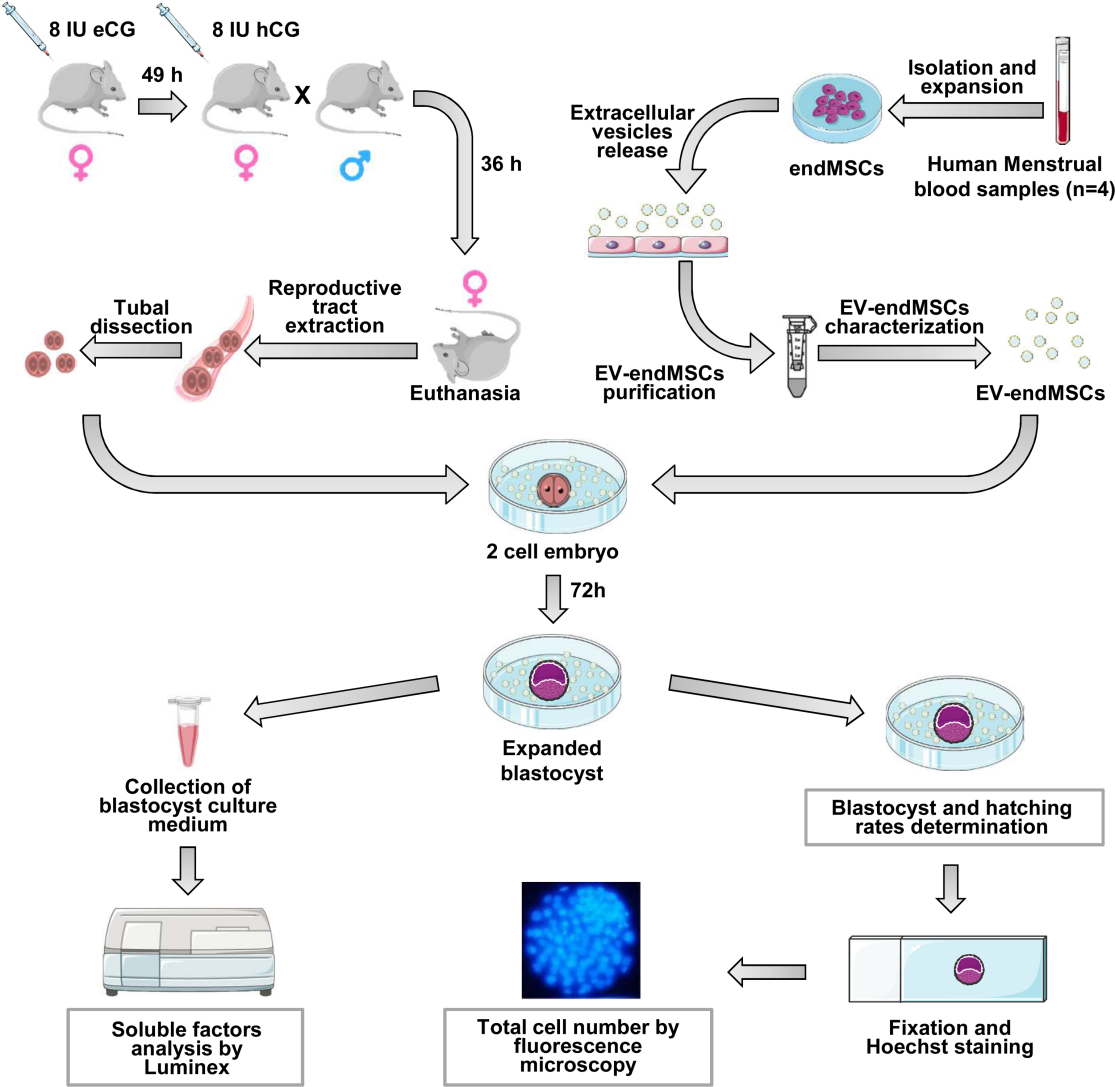
Description of the study and methodological design.

Endometrial Mesenchymal Stromal Cells (endMSCs) were isolated from menstrual blood of four healthy women (Fig 1) according to previously described protocols [20,21]. Inclusion criteria for women were: females without infection, no hormone therapy and ages between 30–40 years. The exclusion criteria were: females with HBV, HCV or HIV infection, immune disorders and under hormone treatments. The samples were collected on day 2 or 3 of the menstrual cycle by the use of a menstrual cup. Written informed consent was obtained from all donors under the auspices of the Minimally Invasive Surgery Centre Research Ethics Committee, which approved this study. Briefly, menstrual blood was diluted 1:2 in PBS and centrifuged at 450 x g for 10 minutes. Supernatants were discarded in order to remove the residues of cervical mucus and cells were re-suspended in DMEM containing 10% fetal bovine serum (FBS), 1% penicillin/streptomycin and 1% glutamine. Cells were seeded onto tissue culture flasks and expanded at 37°C in 95% air and 5% CO_2_ atmosphere. Non-adherent cells were removed after 24h. Adherent cells were cultured to 80% confluency and detached using PBS containing 0.25% trypsin (v/v). Cells were seeded again at a density of 5000 cells/cm^2^. Culture medium was changed every three days.

### Phenotypical and functional characterization of endMSCs

The differentiation assay of endMSCs was performed when the cells reached 80% of confluence. Standard *in vitro* differentiation assays were used to promote osteogenic, adipogenic and chondrogenic differentiation. Cells were cultured for 21 days with differentiation specific media (Stem Pro Adipogenesis, Chondrogenesis and Osteogenesis Differentiation Kits, Gibco, Thermo Fisher Scientific, MA, USA) which was replaced every three days. Cells were stained to evidence adipogenic, chondrogenic and osteogenic differentiation with Oil Red O, Alcian Blue 8GX and Alizarin Red S stains, respectively. Differentiated cells were observed by optical microscopy. The degree of adipogenic, chondrogenic and osteogenic differentiations was determined by extracting the stain with 6M guanidine-HCl (Alcian Blue 8GX and Alizarin Red S stains) or pure isopropanol (Oil Red O stain). The absorbance of the extracts was quantified at 490 nm (Oil Red O and Alizarin Red S stains) and at 600 nm (Alcian Blue 8GX).

For the phenotypic analysis, 2 × 10^5^ endMSCs were stained with human monoclonal antibodies (mAbs) against CD29, CD31, CD34, CD44, CD45, CD49d, CD49f, CD56, CD73, CD90, CD105 and HLA-DR using the appropriate concentrations of mAbs in the presence of PBS containing 2% FBS. The endMSCs and mAbs were incubated for 30 min at 4°C. The cells were washed and re-suspended in PBS. Isotype-matched antibodies were used as negative controls. The flow cytometric analysis was performed on a FACScalibur cytometer (BD Biosciences, CA, USA) after acquisition of 10^5^ events.

Viable cells were selected using forward and side scatter characteristics and fluorescence was analyzed using CellQuest software (BD Biosciences, CA, USA). Isotype-matched negative control antibodies were used in the experiments. The percentage of positive cells above the negative control (isotype controls) was determined. Cells were analyzed at passages 3–4.

### Isolation, purification and characterization of extracellular vesicles from endMSCs

An enriched fraction of endMSCs-derived exosomes, contained in the isolated extracellular vesicles, was obtained from endMSCs cultured in 175 cm^2^ flasks using a previously optimized protocol [22]. When cells reached a confluence of 80%, culture medium (DMEM containing 10% FBS) was replaced by exosome isolation medium (DMEM containing 1% insulin–transferrin–selenium). The endMSCs supernatants were collected every 3–4 days and centrifuged at 1000 x g for 10 min and 5000 x g for 20 min at 4°C to eliminate dead cells and debris. Subsequently, the supernatants were filtered twice using a sterile cellulose acetate filter of 0.45 μm and then on of 0.20 μm (Corning, NY, USA). About 15 ml of these endMSCs supernatants were ultra-filtered through 3kDa MWCO Amicon® Ultra Devices (Merck-Millipore, MA, USA) at 4000 x g for 1 hour at 4°C. The concentrated supernatants were collected and stored at -20°C. Prior to *in vitro* experiments, the concentration of microvesicles was indirectly measured by quantifying the protein content by a Bradford assay (BioRad Laboratories, CA, USA).

The concentration and size of purified EV-endMSCs were quantified by nanoparticle tracking analysis (NanoSight Ltd, Amesbury, UK) equipped with fast video capture that relates the rate of Brownian motion to particle size. Results were analyzed using the particle-tracking analysis software package version 2.2. The equipment configuration for this analysis was: frames processed: 900. 899; frames per second: 30; calibration: 166 nm/pixel; automatic defocus: Auto; detection threshold: 4 Multi; minimum size expected: Auto; tracking minimum size: 100 nm; temperature: 24–28°C; viscosity: 0.80–0.95 cP.

For the flow cytometric analysis by fluorescent activated cells sorting, EV-endMSCs were bounded to latex beads and labeled with fluorophore-conjugated antibodies as described by Théry et al. [23]. Briefly, EV-endMSCs (5 μg of exosomal proteins) were conjugated overnight at 4°C with 10 μl of Aldehyde/Sulfate latex beads, 4% w/v, 4 μm (Molecular Probes, OR, USA). 110 μl of 1M glycine were added to each tube and incubated for 30 minutes. Then the samples were centrifuged and re-suspended in a final volume of 0.5 ml PBS, containing 0.5% bovine serum albumin (BSA; w/v). These EV-endMSCs-coated beads were incubated for 30 minutes at 4°C with anti-CD9 and anti-CD63 human monoclonal antibodies (BD Biosciences, CA, USA). The EV-endMSCs -coated beads were washed and re-suspended in PBS+BSA. The flow cytometry analysis was performed on a FACScalibur cytometer (BD Biosciences, CA, USA) after acquisition of 10^5^ events. Isotype-matched negative control antibodies were used in all the experiments. The percentage of positive cells above the negative control (isotype controls) was determined.

### Soluble factor analysis

At the third day of embryo culture, the embryos were fixed as described below and the cell culture media were individually collected and stored at -20°C for later soluble factors analyses (Fig 1). As negative controls, KSOM medium with or without EV-endMSCs were used and their quantifications were subtracted to the corresponding study groups. The murine-specific soluble factors analyzed were chosen for their importance in the first phases of embryo development: GM-CSF, VEGF, IGF-I, IL-6, M-CSF, EGF and PDGF-AA. These soluble factors were analyzed using a bead-based Magnetic multiplexed Luminex Assay (LXSAMSM, R&D systems, MN, USA) according to the manufacturer’s instructions. The concentrations of the different factors were expressed as pg/ml.

### Animals and superovulation protocol

All the experimental procedures were reviewed and approved by the Ethical Committee of the Jesús Usón Minimally Invasive Surgery Centre. B6D2F1 mice were housed under a 12 h light/12 h dark cycle, at a controlled temperature (19–23°C) with free access to food and water. Females were intraperitoneally (IP) injected with 8 IU of equine chorionic gonadotropin (eCG, Veterin Corion, Divasa Farmavic) followed 49 h later by 8 IU of IP human chorionic gonadotropin (hCG, Foligon, MSD) to trigger ovulation (Fig 1).

### *In vivo* embryo recovery and culture

Female mice (8–12 weeks old) were hormonally stimulated to trigger ovulation as previously described; after hCG injection, females were paired with B6D2 males in a 1:1 ratio. After 36 hours from the hCG injection, females were sacrificed by cervical dislocation and the embryos were collected from the oviducts in M2 (Sigma-Aldrich, Barcelona, spain); these 2-cell embryos were washed in fresh KSOM (Merck-Millipore, Madrid, Spain) and placed in 100 μl droplets (9–12 embryos/droplet) devoid of EV-endMSCs (control) or in KSOM droplets added with 10, 20, 40 or 80 μg/ml of EV-endMSCs from the four different donors individually and media was not replaced throughout the entire culture; embryos were incubated in a 5% CO_2_/95% air atmosphere at 37°C and 100% humidity. For each experiment, 2 cell murine embryos were obtained from 3 different females (36 hours post-hCG) and pooled prior embryo culture (12 different females used in total). The number of initial 2 cell embryos and the percentage of embryos reaching the expanded blastocyst stage as well as embryo hatching after 75 hours in culture were recorded (Fig 1).

### Total cell number

The number of cells in an embryo is a well-known indicator of embryo viability and quality [24]. Therefore, in view of the previous data, after embryo hatching assessment, the blastocysts obtained were fixed in 4% formaldehyde in PBS added with 0.01% of polyvinyl alcohol (PVA; w/v) at 4°C for 12 hours and stained with 2.5 μg/ml of Hoechst 33342 (Eugene, OR, USA) in PBS added with PVA for 10 minutes at 37°C. Then, the blastocysts were mounted on glass slides with glycerol, covered with coverslips and sealed. Embryos were then visualized (Fig 1) using a fluorescence microscope (Nikon Elipse TE2000-S) equipped with an ultraviolet lamp. Cell number was analyzed using the Fiji Image-J Software (1.45q, Wayne Rasband, NIH, USA).

### Statistical analysis

For total cell number analysis data were tested for normality using a Shapiro–Wilk test; results are reported as mean ± standard deviation (SD). Groups were compared using a one way ANOVA due their Gaussian distribution and homoscedasticity. When statistically significant differences were found, a Bonferroni post-hoc test was used to compare pairs of values. Blastocyst and hatching rates were compared among groups by Chi-square test with the Yates correction for continuity. The Fisher’s Exact Test was used when a value of less than 5 was expected in any treatment. A Student t-test for paired comparisons was performed on VEGF and PDGF-AA measurements. The correlation between VEGF and PDGF-AA was calculated using the Pearson correlation coefficient. Statistical analyses were performed using Sigma Plot software version 12.3 for Windows (Systat Software, IL, USA) or with SPSS-21 software (SPSS, IL, USA); p < 0.05 was considered as statistically significant.

## Results

### Phenotypic profile of endMSCs

**Fig 2:**
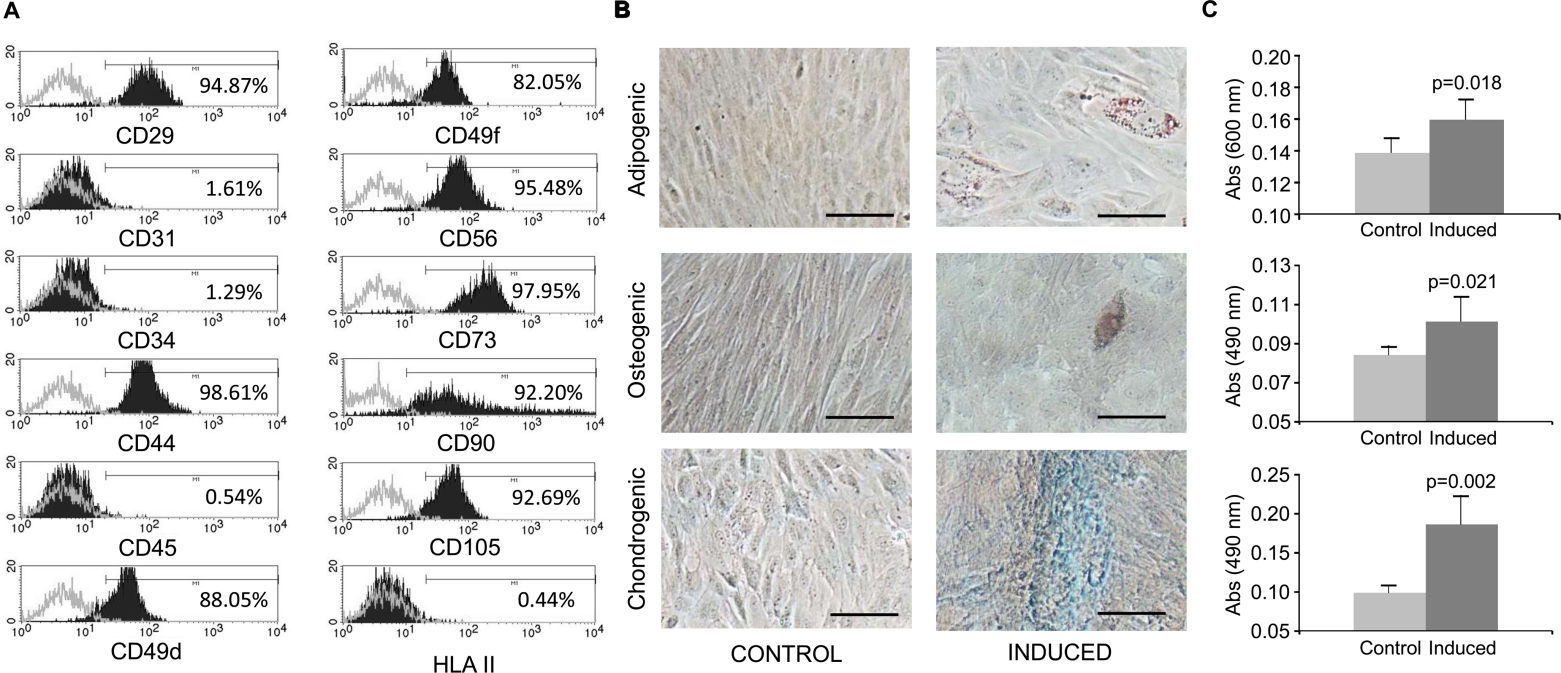
Phenotypical characterization and differentiation potential of endMSCs. (A) Flow cytometry analysis of endMSCs isolated from menstrual blood. A representative histogram together with the expression levels is shown. The expression of cell surface markers is represented as the percentage of positive cells (black lined histogram) above the negative control (grey lined histogram). (B) *In vitro* differentiation potential of endMSCs. The cells were *in vitro* cultured for 21 days with standard medium (Control) or with specific differentiation media for adipogenic, osteogenic and chondrogenic lineages (Induced). Differentiation towards adipocytes, osteocytes and chondrocytes was evidenced by Oil Red O, Alizarin Red and Alcian Blue 8GX respectively. Scale bar: 100 μm. (C) The adipogenic (above), osteogenic (middle) and chondrogenic (below) differentiation degree was quantified by determining the absorbance of the extracts at 490 nm (Oil Red O and Alizarin Red S staining) and at 600 nm (Alcian Blue 8GX). Four independent experiments using four different cell lines were performed and a Mann-Whitney U test was used. p-values are shown in the figure.

The phenotypic analysis of endMSCs cultures was carried out by flow cytometry. As reported by the representative histograms (Fig 2A), endMSCs were negative for CD31, CD34, CD45, HLA-DR and positive for the stemness markers CD29, CD44, CD49d, CD49f, CD56, CD73, CD90 and CD105. The differentiation towards the adipogenic, chondrogenic and osteogenic lineages was confirmed by microscopic analysis (Fig 2B) and stain quantification (Fig 2C).

### Size distribution, concentration and exosome specific markers in EV-endMSCs

**Fig 3:**
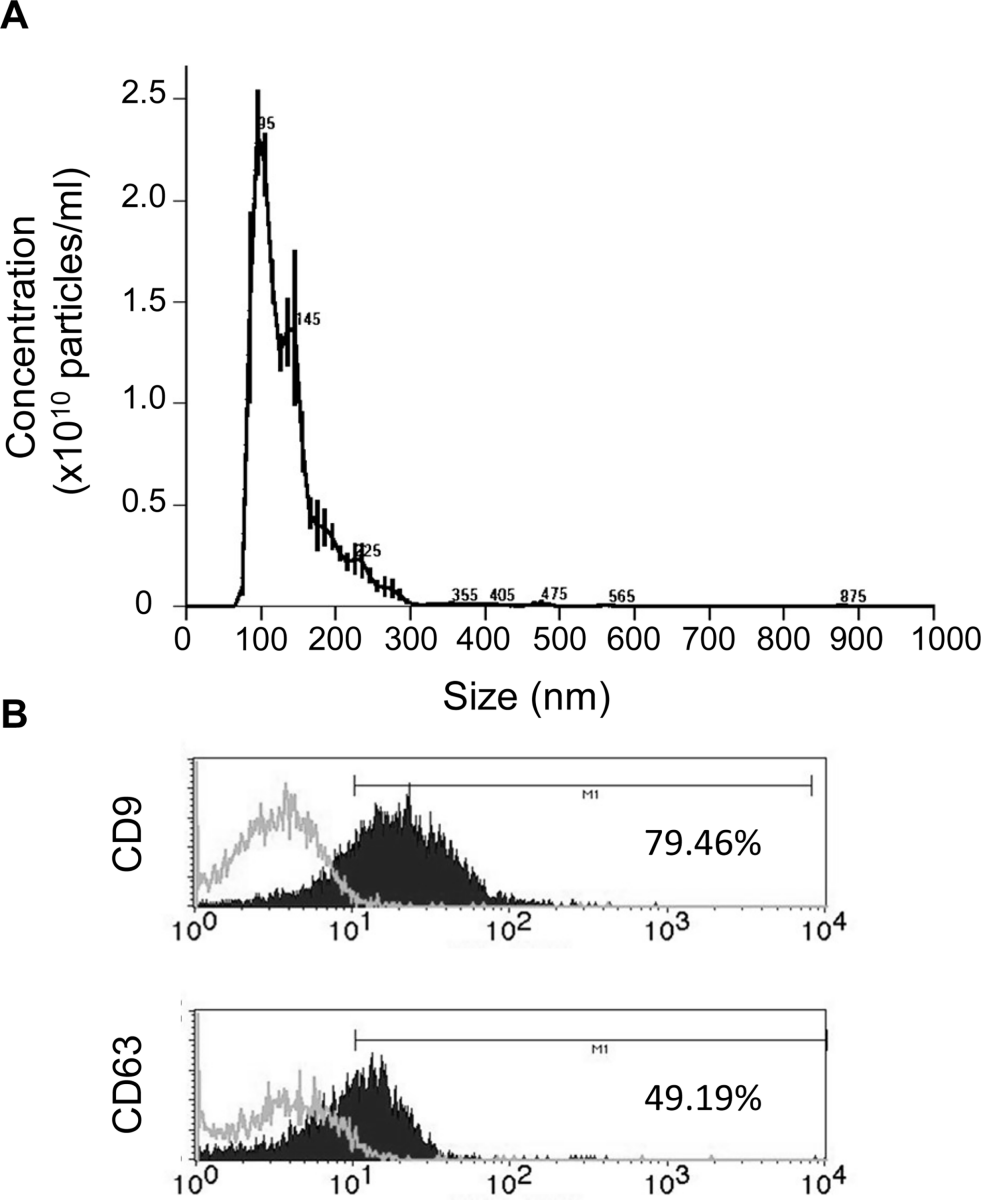
Characterization of EV-endMSCs. (A) Representative graph of nanoparticle tracking analysis to quantify size distribution and particle concentration of EV-endMSCs. (B) Flow cytometry analysis on EV-endMSCs for exosomal markers CD9 and CD63. A representative histogram together with the expression levels is shown. The expression of cell surface markers is represented as as the percentage of positive cells (black lined histogram) above the negative control (grey lined histogram).

In order to quantify the proteins in the enriched fraction of exosomes, a Bradford assay was performed ranging their protein concentrations between 350 and 750 μg/ml. The nanoparticle tracking analysis of EV-endMSCs revealed that the mean size and standard deviation of isolated vesicles from four different donors was 153.5±63.05 nm, while their concentration was 3.31x10^11^ ± 3.8x10^9^ particles/ml. Fig 3A shows a representative analysis of nanoparticle tracking analysis. Finally, the EV-endMSCs were phenotypically characterized by flow cytometry with specific exosomal markers. The analysis of CD9 and CD63 demonstrated a positive expression of these exosome-related proteins (Fig 3B).

### Development to the blastocyst stage, embryo hatching and total cell number count

**Fig 4:**
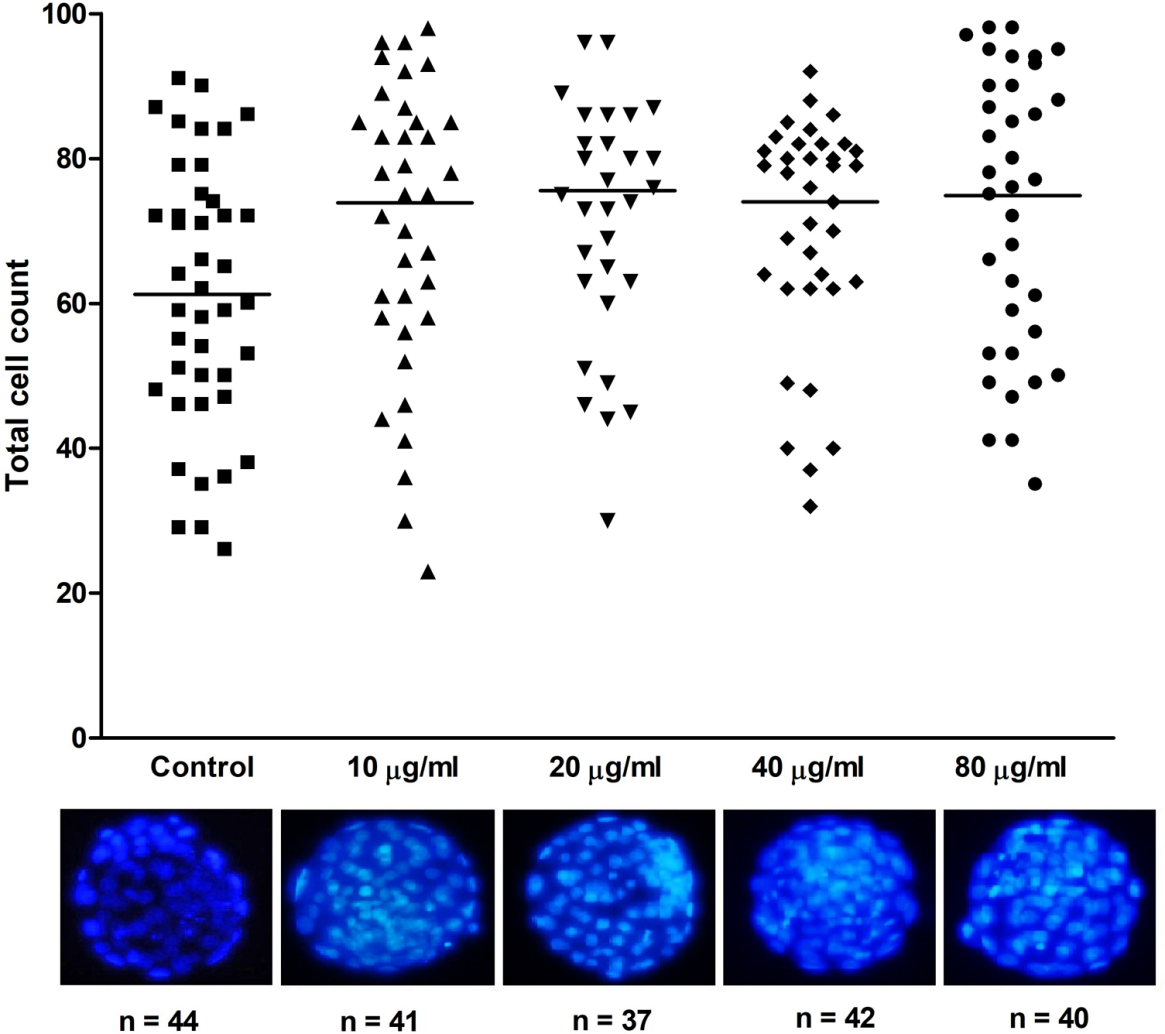
Total cell number of murine blastocysts cultured in presence or absence of EV-endMSCs. Total cell number of the obtained expanded blastocysts was obtained after Hoechst 33342 staining and subsequent evaluation by fluorescence microscopy. For each treatment, the individual blastomere count is represented. Horizontal bars show the mean values. All the treatments differ statistically from the control (p<0.05). Representative micrographs of expanded blastocysts cultured with varying dosages of EV-endMSCs are shown.

Our results showed that the developmental competence of the embryos did not vary disregarding EV-endMSCs addition or the dosage used. The blastocyst rate of the control embryos devoid of EV-endMSCs was 86.8%, this percentage was 98.2% for the 10 μg/ml dose, 92.9% for 20 μg/ml, 79.6% when 40 μg/ml of EV-endMSCs were added and 84.9% for the 80 μg/ml dose and no statistically significant differences were observed between groups (p>0.05; Table 1). Conversely, total cell number was significantly enhanced ranging from 73.8–75.6 cells/embryo (37–41 blastocysts evaluated per treatment) when EV-endMSCs were added exceeding the non-EV-endMSCs added control (61.2 ± 19.6 cells/embryo, n = 44; p<0.05), demonstrating that EV-endMSCs significantly promoted blastomere division during embryonic development (Fig 4). Furthermore, as seen in Table 1, embryo hatching was consistently enhanced for all the EV-endMSCs dosages tested, although statistically significant differences were only observed between the control (20.5% hatching embryos) and the 20 μg/ml and 40 μg/ml EV-endMSCs dosages (54.1 and 47.6% of hatching embryos respectively; p<0.05).

**Table 1:** Embryo development to the blastocyst stage and hatching rates.

Treatment	Initial 2 cell embryos	Blastocyst (%)	Hatching (%)
Control	53	86.8^a^	20.5^a^
10 μg/ml EV-endMSCs	56	98.2^a^	39.0^a,b^
20 μg/ml EV-endMSCs	57	92.9^a^	54.1^b^
40 μg/ml EV-endMSCs	49	79.6^a^	47.6^b^
80 μg/ml EV-endMSCs	53	84.9^a^	40^a,b^

The initial number of 2 cell embryos retrieved in uterus (n) as well as blasocyst rate in % and hatching embryos in % is provided (hatching rates were calculated as the number of blastocysts that hatched/total blastocyst number per treatment); different superscripts represent statistically significant differences (p<0.05).

### Quantification of soluble factors secreted during embryo development *in vitro*

**Fig 5:**
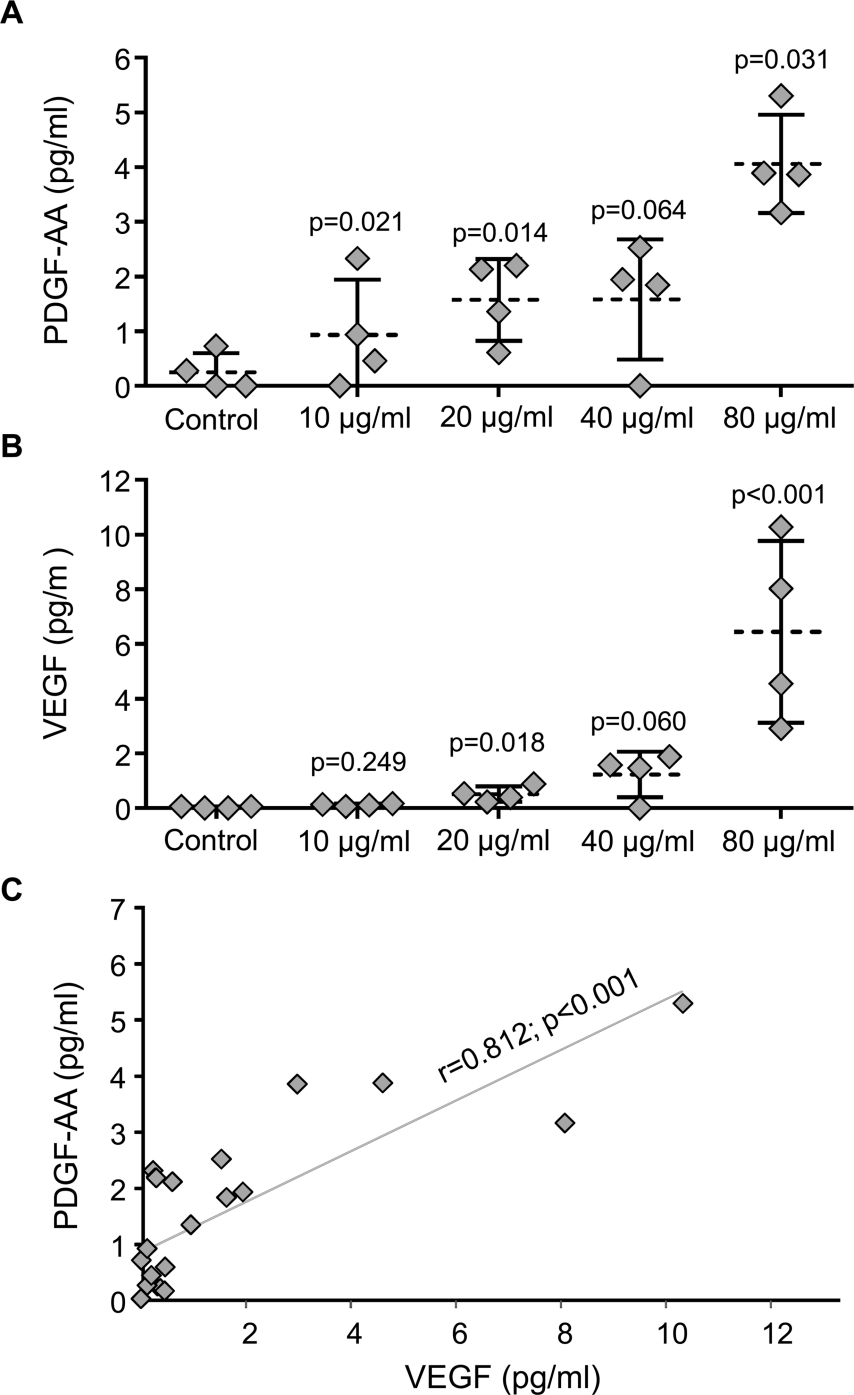
Quantification of VEGF and PDGF-AA secreted during embryo culture. Soluble factors released by the developing embryos co-cultured with EV-endMSCS were quantified by the Luminex xMAP technology at the third day of embryo culture. (A) PDGF-AA secreted by blastocyst embryos. All the data were compared by Student t-test for paired comparisons with respect to control group. The mean (dotted line) ±SD from four independent experiments, as well as individual measures (rhombuses), are shown. (B) VEGF secreted by blastocyst embryos. All the data were compared by Student t-test for paired comparisons with respect to control group. The mean (dotted line) ±SD from four independent experiments, as well as individual measures (rhombuses), are shown. (C) Correlation between PDGF-AA and VEGF. Correlation line as well as individual measures (rhombuses) are represented. The Pearson correlation coefficient (r) together with its significance level (p) is shown.

The analysis of soluble factors was performed at the third day of embryo culture. Unfortunately, the analysis of murine molecules: GM-CSF, IGF-I, IL-6, M-CSF and EGF were below the detection limits of the technique and the quantification of these molecules was impossible at this time point (data not acquired). On the contrary, blastocyst-released PDGF-AA and VEGF were detectable in all supernatants at day 3 of embryo culture for all the different EV-endMSCs dosages tested. Our results demonstrated an EV-endMSCs concentration-dependent increase of VEGF and PDGF-AA released by the embryos (Fig 5). It is important to note that the hypothetical presence of VEGF or PDGF-AA factors from EV-endMSCs was considered and negative controls (culture medium with/without EV-endMSCs) were included for the different samples and this background was subtracted for each sample. However, as we could not discard cross-reactivity between human and mouse PDGF-AA in the Luminex assay, there is a possibility that residual human PDGF-AA with exosomal origin coming from embryos could have interfered in the measurements. The separated values of the experimental samples and negative control samples are shown in S1 Fig.

In the case of PDGF-AA, significant differences were found between negative controls (embryos cultured in the absence of EV-endMSCs) and embryos co-cultured with EV-endMSCs. The EV-endMSCs added at 10 μg/ml, 20 μg/ml, 40 μg/ml and 80 μg/ml showed the following p values: p = 0.021, p = 0.014, p = 0.064 and p = 0.031 respectively (Fig 5A).

In the case of VEGF, several significant differences were found between negative controls (blastocysts cultured without EV-endMSCs) and blastocysts co-cultured with EV-endMSCs. The EV-endMSCs supplemented at 10 μg/ml, 20 μg/ml, 40 μg/ml and 80 μg/ml showed the following p values: p = 0.249, p = 0.018, p = 0.060 and P<0.001 respectively (Fig 5B).

Finally, as shown in Fig 5C, our results demonstrated that the released VEGF and PDGF-AA produced by the blastocyst during their development *in vitro* were very significantly correlated (r = 0.812, p<0.001).

## Discussion

Exosomes derived from MSCs are a matter of study as they are known to exert a therapeutic role in different physiological and pathological conditions. In the case of endMSCs, these cells participate in tissue remodeling which is essential for the endometrium [25] exhibiting immunomodulatory potential through the release of soluble factors [26]. These cells can be easily obtained during menstruation and their expansion, characterization and subsequent exosome retrieval can be achieved in the laboratory. Specifically, human exosomes released in the uterus have been demonstrated to profoundly influence implantation and embryo-maternal crosstalk [8].

Additionally, it has been demonstrated that *in vitro* produced bovine embryos release exosomes and that their composition varies depending on the embryo competence [27]; on the other hand it has been shown that, when the culture medium is not replaced during the entire embryo culture, the blastocyst rate, total cell number and calving significantly improve in bovine cloned embryos [28]. These effects have been related to exosome secretion by the embryos in the culture medium as removal of these exosomes by medium replacement impairs embryo development, while its supplementation to exosome-depleted embryos increases embryo quality [28]. Taken together, these reports reflect the complex interplay existing between the maternal environment and the embryo as exosome release and uptake determines embryo competence, quality and birth rate.

Even when exosomal content is still under study, what it is already known is that exosomes are locally released and are meant to interact and transfer their cargo to the target cells. Although the exact mechanisms are under study, in the case of the uterine environment these mechanisms are even more intricate, as the exosome content also depends on the hormonal environment and gestational status [29,30]. Several studies have been conducted to understand their *in vitro* features such as protein cargo [8] or micro RNA content [31] although the role that they may play *in vivo* or the optimal dosage to be used remains unexplored. Based on the previously mentioned literature, we aimed to elucidate if human EV-endMSCs influenced the soluble factors released by blastocyst, the development and hatching of blastocysts or the blastomere proliferation of murine embryos obtained *in vivo*. Our results did not evidence any effect of EV-endMSCs on embryo development as the blastocyst rates remained unchanged between treatments (over 79% blastocyst rate for all the treatments tested). These data are in agreement with previous reports in the bovine species in which exosomes derived from homologous oviductal explants did not increase the blastocyst yield [32,33]. However, core differences exist in both experimental settings as bovine *in vitro* produced embryos exhibit consistently lower developmental competence than B6D2F1 murine *in vivo* obtained two cell embryos [34]. Nevertheless, our results do not evidence any toxic effect of the EV-endMSCs dosages tested, and thus, the ones used in the present study can be considered non-toxic. As embryo development to the blastocyst stage does not predict embryo quality, the expanded blastocysts obtained were fixed and stained with Hoechst 33342 in order to determine their quality by total blastomere counts [35]. Interestingly, our results demonstrated an increase in blastomere count at all the EV-endMSCs dosages tested over the control demonstrating a positive effect of these microvesicles on embryonic total cell number. However, embryo transfer will be performed in next studies to rule out an increased incidence of embryonic aneuploidies in embryos supplemented with EV-endMSCs, although their incidence is very low in this species [36]. As previously mentioned, homologous oviductal exosomes increase the quality of bovine embryos in terms of survival rate after cryopreservation and gene expression [32,33]. In addition, exosomes derived from cloned bovine embryos also enhanced total cell number and the inner cell mass/trophoectoderm cell of the embryos [28] and these results parallel the ones obtained in the present work. To further confirm that EV-endMSCs increase embryo quality *in vitro*, hatching rates were also evaluated. Interestingly, statistically significant differences in hatching rates were only detected in the 20 and 40 μg/ml dosages compared against control, although for the 10 and 80 μg/ml dosages hatching rates were almost doubled compared to the non-microvesicles added treatment. It is known that embryo hatching in the mouse is achieved by an embryonic-mediated lytic mechanism and by the pressure of the expanding blastocele/blastocyst against the zona [37]. However, the exact mechanism of hatching is still controversial as some authors claim that the lytic mechanism is predominant and the blastocyst pressure is less relevant [38]; thus our results parallel those of Gordon *et al*. who described that enzymatic digestion of the zona pellucida using Tyrode’s solution better induces embryo hatching compared to mineral injection under the zona to mimic the mechanical embryo pressure as, even when total cell number significantly increases in each EV-endMSCs treatment, hatching rates improve significantly only for the 20 and 40 μg/ml dosages. These results reflect that the 10 μg/ml is a low dose while with the 80 μg/ml dosage we may be working at saturation conditions; therefore the 20–40 μg/ml dosages seem to be adequate reference ranges to work with. Our preliminary proteomic analyses of the EV-endMSCs have revealed a wide range of proteins closely related to embryo development and implantation (manuscript in preparation). As an example, transferrin [39], vinculin [40], and fibronectin [41,42] have been demonstrated to promote embryo development being essential in the early embryo development and survival. Thus, EV-endMSC could be promoting embryo blastomere division and hatching by the specific protein cargo released to the embryo culture medium. In addition, core proteins related to embryo implantation such as matrix metalloproteinase-2, -3 and -9 [43,44], or E-cadherin [45] were also identified. In summary, according to the proteomic profile of EV-endMSCs and the enhanced hatching observed in our EV-endMSCs embryos (positively related with embryo implantation [46]), our results may indicate a higher implantation potential of the obtained embryos, but this theory has to be further confirmed.

Apart from the analysis of embryo developmental competence, total blastomere count and blastocyst/hatching rate; we aimed to evaluate the release of soluble factors by murine embryos. It is known that embryos release cytokines that play a role in embryonic development, embryo-maternal recognition and maintenance of the proper hormonal environment [47]. However, the exact molecules that modulate the development of the pre-implantation embryo still remains a matter of study [48]. In this study, our analysis was firstly focused on the quantification of GM-CSF which has been demonstrated to promote blastocyst development [49], embryo implantation [50] as well as embryo survival [51]. Unfortunately, in our experimental conditions, the quantification analysis by Luminex xMAP detection was not sensitive enough to quantify murine GM-CSF. In fact, the detection limit of our Luminex assay was 11.65 pg/ml, while the detection limit by commercially available ELISAs is usually between 4 to 5 pg/ml. Based on that, future studies for quantifying murine GM-CSF will be performed by ELISA tests or by using blastocysts supernatants at different time points. Similarly to murine GM-CSF, the analyses of murine IL-6, M-CSF, EGF and IGF-I were also below the detection limit. Previous studies in human embryo culture-conditioned media have demonstrated that IL-6 was undetectable in embryo supernatants [52]. However, we should not rule out that ELISA (with better detection limit: 24.56 pg/ml for Luminex *vs*. 7–8 pg/ml for ELISA), may provide a more reliable quantitative analysis of this cytokine which has been found to be secreted by trophoblast cells [53]. In the case of IGF-I and EGF, these two growth factors were also undetectable in our experimental conditions. The IGF-I has been reported to promote blastocyst development [49] and is positively correlated with embryo quality when present in high concentrations in the follicular fluid [54]. In the case of EGF, this molecule stimulates trophoblast development having a key role in the implantation process [55,56].

Although several soluble molecules were undetectable in the culture medium of murine embryos, detectable levels were observed for VEGF and PDGF-AA. VEGF is intensely synthesized by blastocysts during embryo development in humans [57] and PDGF-AA has been associated to enhanced embryo quality and developmental potential [58]. In our experimental conditions, the addition of EV-endMSCs to embryo cell culture triggered the release of these two growth factors and this release was EV-endMSCs concentration-dependent.

In the case of VEGF, the synthesis of this molecule has been initially described during embryonic angiogenesis [59] and seems to be the responsible of vascularization in placenta and decidua when secreted by trophoblastic giant cells [60]. In fact, the angiogenic potential of exosomes from different origins (human placenta-derived MSCs [61], umbilical cord blood [62] endothelial cells [63], human-induced pluripotent stem cell-derived MSCs [64] or bone marrow derived MSCs [65], among others) has been reported. Finally, and similarly to our experimental conditions, in mouse models, the role of VEGF has been associated with embryo implantation and embryonic vasculogenesis [66].

Regarding PDGF-AA and embryo development, the first expression analysis demonstrating the expression of PDGF-AA in embryonic murine cells [67]. This molecule has been linked with early embryo development and more recently, it has been associated with the regulation of programmed cell death mediating the fine-tuning formation of the primitive endoderm at the end of the preimplantation period [68]. In general, PDGF-AA has been defined as a mitogenic factor driving the proliferation of undifferentiated cells [69] and in later maturation stages it has been associated with cell differentiation, tissue remodeling, patterning and morphogenesis [70].

Interestingly, even when the effect of human EV-endMSCs was tested on murine embryos, the embryos increased the secretion of VEGF and PDGF-AA of murine origin. This fact highlights that EV-endMSCs exert their effect in a non-species-specific manner and suggest that the murine model can be a good candidate to further investigate the efficacy of EV-endMSCs of human origin on these embryos.

To summarize, to the authors’ best knowledge, this is the first report describing the lack of toxicity and beneficial effect of human EV-endMSCs on embryos of any species. The increased release of VEGF and PDGF-AA may indicate that the beneficial effect could be mediating not only an enhanced embryo quality reflected by a significant increase in total cell number per blastocyst and embryo hatching, but also supporting angiogenesis, vascularization, differentiation and tissue remodeling of the endometrium after embryo hatching in view of the soluble factors released. These results confirm a beneficial effect of EV-endMSCs in the field of assisted reproduction and aim to impulse future research in this still underexplored area.

## Supporting information

S1 Fig Quantification of VEGF and PDGF-AA secreted during embryo culture. VEGF (A) and PDF-AA (B) concentrations measured by Luminex in experimental samples (box and whiskers diagrams, corresponding to embryos exposed to different concentrations of EV-endMSCs) and negative control samples used as background (plot diagrams, corresponding to culture medium with different concentrations of EV-endMSCs). The means±SD from four independent experiments are shown.
(TIF)

## References

[1] Khoury M, Alcayaga-Miranda F, Illanes SE, Figueroa FE. The promising potential of menstrual stem cells for antenatal diagnosis and cell therapy. Front Immunol. 2014;5: 205 doi: 10.3389/fimmu.2014.00205 24904569 10.3389/fimmu.2014.00205 PMC4032935

[2] Uder C, Brückner S, Winkler S, Tautenhahn H-M, Christ B. Mammalian MSC from selected species: Features and applications. Cytometry A. 2018;93: 32–49. doi: 10.1002/cyto.a.23239 28906582 10.1002/cyto.a.23239

[3] Burke J, Hunter M, Kolhe R, Isales C, Hamrick M, Fulzele S. Therapeutic potential of mesenchymal stem cell based therapy for osteoarthritis. Clin Transl Med. 2016;5: 27 doi: 10.1186/s40169-016-0112-7 27510262 10.1186/s40169-016-0112-7 PMC4980326

[4] Volarevic V, Nurkovic J, Arsenijevic N, Stojkovic M. Concise review: Therapeutic potential of mesenchymal stem cells for the treatment of acute liver failure and cirrhosis. Stem Cells. 2014;32: 2818–2823. doi: 10.1002/stem.1818 25154380 10.1002/stem.1818

[5] Rani S, Ryan AE, Griffin MD, Ritter T. Mesenchymal Stem Cell-derived Extracellular Vesicles: Toward Cell-free Therapeutic Applications. Mol Ther. 2015;23: 812–823. doi: 10.1038/mt.2015.44 25868399 10.1038/mt.2015.44 PMC4427881

[6] Théry C, Zitvogel L, Amigorena S. Exosomes: composition, biogenesis and function. Nat Rev Immunol. 2002;2: 569–579. doi: 10.1038/nri855 12154376 10.1038/nri855

[7] Wang L, Yu Z, Wan S, Wu F, Chen W, Zhang B, et al Exosomes Derived from Dendritic Cells Treated with Schistosoma japonicum Soluble Egg Antigen Attenuate DSS-Induced Colitis. Front Pharmacol. 2017;8: 651 doi: 10.3389/fphar.2017.00651 28959207 10.3389/fphar.2017.00651 PMC5603738

[8] Greening DW, Nguyen HPT, Elgass K, Simpson RJ, Salamonsen LA. Human Endometrial Exosomes Contain Hormone-Specific Cargo Modulating Trophoblast Adhesive Capacity: Insights into Endometrial-Embryo Interactions. Biol Reprod. 2016;94: 38 doi: 10.1095/biolreprod.115.134890 26764347 10.1095/biolreprod.115.134890

[9] Mutlu L, Hufnagel D, Taylor HS. The endometrium as a source of mesenchymal stem cells for regenerative medicine. Biol Reprod. 2015;92: 138 doi: 10.1095/biolreprod.114.126771 25904012 10.1095/biolreprod.114.126771 PMC4652610

[10] Chen L, Xiang B, Wang X, Xiang C. Exosomes derived from human menstrual blood-derived stem cells alleviate fulminant hepatic failure. Stem Cell Res Ther. 2017;8: 9 doi: 10.1186/s13287-016-0453-6 28115012 10.1186/s13287-016-0453-6 PMC5260032

[11] Alcayaga-Miranda F, González PL, Lopez-Verrilli A, Varas-Godoy M, Aguila-Díaz C, Contreras L, et al Prostate tumor-induced angiogenesis is blocked by exosomes derived from menstrual stem cells through the inhibition of reactive oxygen species. Oncotarget. 2016;7: 44462–44477. doi: 10.18632/oncotarget.9852 27286448 10.18632/oncotarget.9852 PMC5190111

[12] Homer H, Rice GE, Salomon C. Review: Embryo- and endometrium-derived exosomes and their potential role in assisted reproductive treatments-liquid biopsies for endometrial receptivity. Placenta. 2017;54: 89–94. doi: 10.1016/j.placenta.2016.12.011 28043656 10.1016/j.placenta.2016.12.011

[13] Chaouat G, Dubanchet S, Ledée N. Cytokines: Important for implantation? J Assist Reprod Genet. 2007;24: 491–505. doi: 10.1007/s10815-007-9142-9 18044017 10.1007/s10815-007-9142-9 PMC3455031

[14] Salamonsen LA, Evans J, Nguyen HPT, Edgell TA. The Microenvironment of Human Implantation: Determinant of Reproductive Success. Am J Reprod Immunol. 2016;75: 218–225. doi: 10.1111/aji.12450 26661899 10.1111/aji.12450

[15] Cui W, Mager J. Transcriptional Regulation and Genes Involved in First Lineage Specification During Preimplantation Development. Adv Anat Embryol Cell Biol. 2018;229: 31–46. doi: 10.1007/978-3-319-63187-5_4 29177763 10.1007/978-3-319-63187-5_4 PMC7558833

[16] Marcho C, Cui W, Mager J. Epigenetic dynamics during preimplantation development. Reproduction. 2015;150: R109–120. doi: 10.1530/REP-15-0180 26031750 10.1530/REP-15-0180 PMC4529766

[17] Johnson MH. From mouse egg to mouse embryo: polarities, axes, and tissues. Annu Rev Cell Dev Biol. 2009;25: 483–512. doi: 10.1146/annurev.cellbio.042308.113348 19575654 10.1146/annurev.cellbio.042308.113348

[18] Kim HJ, Park SB, Yang JB, Choi YB, Lee KH. Effects of laser-assisted hatching and exposure time to vitrification solution on mouse embryo development. Clin Exp Reprod Med. 2017;44: 193–200. doi: 10.5653/cerm.2017.44.4.193 29376016 10.5653/cerm.2017.44.4.193 PMC5783916

[19] Park SB, Kim HJ, Choi YB, Ahn KH, Lee KH, Yang JB, et al The effect of various assisted hatching techniques on the mouse early embryo development. Clin Exp Reprod Med. 2014;41: 68–74. doi: 10.5653/cerm.2014.41.2.68 25045630 10.5653/cerm.2014.41.2.68 PMC4102692

[20] Moreno R, Rojas LA, Villellas FV, Soriano VC, García-Castro J, Fajardo CA, et al Human Menstrual Blood-Derived Mesenchymal Stem Cells as Potential Cell Carriers for Oncolytic Adenovirus. Stem Cells Int. 2017;2017 doi: 10.1155/2017/3615729 28781596 10.1155/2017/3615729 PMC5525077

[21] Zemel’ko VI, Kozhukharova IB, Alekseenko LL, Domnina AP, Reshetnikova GF, Puzanov MV, et al Neurogenic potential of human mesenchymal stem cells isolated from bone marrow, adipose tissue and endometrium: a comparative study. Tsitologiia. 2013;55: 101–110. 23718072

[22] Álvarez V, Blázquez R, Sánchez-Margallo FM, DelaRosa O, Jorge I, Tapia A, et al Comparative study of isolated human mesenchymal stem cell derived exosomes for clinical use. Acta bioquim clin latinoam. 2015;49: 311–320.

[23] Théry C, Amigorena S, Raposo G, Clayton A. Isolation and characterization of exosomes from cell culture supernatants and biological fluids. Curr Protoc Cell Biol. 2006;3.22: 1–29. doi: 10.1002/0471143030.cb0322s30 18228490 10.1002/0471143030.cb0322s30

[24] Alpha Scientist in Reproductive Medicine and ESHRE Special Interest Group of Embryology. The Istanbul consensus workshop on embryo assessment: proceedings of an expert meeting. Hum Reprod. 2011;26: 1270–1283. doi: 10.1093/humrep/der037 21502182 10.1093/humrep/der037

[25] Elsheikh E, Sylvén C, Ericzon B-G, Palmblad J, Mints M. Cyclic variability of stromal cell-derived factor-1 and endothelial progenitor cells during the menstrual cycle. Int J Mol Med. 2011;27: 221–226. doi: 10.3892/ijmm.2010.570 21132258 10.3892/ijmm.2010.570

[26] Wang H, Jin P, Sabatino M, Ren J, Civini S, Bogin V, et al Comparison of endometrial regenerative cells and bone marrow stromal cells. J Transl Med. 2012;10: 207 doi: 10.1186/1479-5876-10-207 23038994 10.1186/1479-5876-10-207 PMC3504519

[27] Mellisho EA, Velásquez AE, Nuñez MJ, Cabezas JG, Cueto JA, Fader C, et al Identification and characteristics of extracellular vesicles from bovine blastocysts produced in vitro. PLoS ONE. 2017;12: e0178306 doi: 10.1371/journal.pone.0178306 28542562 10.1371/journal.pone.0178306 PMC5444795

[28] Qu P, Qing S, Liu R, Qin H, Wang W, Qiao F, et al Effects of embryo-derived exosomes on the development of bovine cloned embryos. PLoS ONE. 2017;12: e0174535 doi: 10.1371/journal.pone.0174535 28350875 10.1371/journal.pone.0174535 PMC5370134

[29] Burns G, Brooks K, Wildung M, Navakanitworakul R, Christenson LK, Spencer TE. Extracellular Vesicles in Luminal Fluid of the Ovine Uterus. Ye X, editor. PLoS ONE. 2014;9: e90913 doi: 10.1371/journal.pone.0090913 24614226 10.1371/journal.pone.0090913 PMC3948691

[30] Nguyen HPT, Simpson RJ, Salamonsen LA, Greening DW. Extracellular Vesicles in the Intrauterine Environment: Challenges and Potential Functions. Biology of Reproduction. 2016;95: 109–109. doi: 10.1095/biolreprod.116.143503 27655784 10.1095/biolreprod.116.143503 PMC5333933

[31] Altmäe S, Koel M, Võsa U, Adler P, Suhorutšenko M, Laisk-Podar T, et al Meta-signature of human endometrial receptivity: a meta-analysis and validation study of transcriptomic biomarkers. Sci Rep. 2017;7: 10077 doi: 10.1038/s41598-017-10098-3 28855728 10.1038/s41598-017-10098-3 PMC5577343

[32] Lopera-Vasquez R, Hamdi M, Maillo V, Gutierrez-Adan A, Bermejo-Alvarez P, Ramírez MÁ, et al Effect of bovine oviductal extracellular vesicles on embryo development and quality *in vitro*. Reproduction. 2017;153: 461–470. doi: 10.1530/REP-16-0384 28104825 10.1530/REP-16-0384

[33] Lopera-Vásquez R, Hamdi M, Fernandez-Fuertes B, Maillo V, Beltrán-Breña P, Calle A, et al Extracellular Vesicles from BOEC in In Vitro Embryo Development and Quality. Sturmey R, editor. PLOS ONE. 2016;11: e0148083 doi: 10.1371/journal.pone.0148083 26845570 10.1371/journal.pone.0148083 PMC4742056

[34] Gu Y, Li Y, Huang X, Zheng J, Yang J, Diao H, et al Reproductive Effects of Two Neonicotinoid Insecticides on Mouse Sperm Function and Early Embryonic Development In Vitro. Wang H, editor. PLoS ONE. 2013;8: e70112 doi: 10.1371/journal.pone.0070112 23922925 10.1371/journal.pone.0070112 PMC3726447

[35] Xu J, Cheung T., Chan ST, Ho P., Yeung WS. Human oviductal cells reduce the incidence of apoptosis in cocultured mouse embryos. Fertility and Sterility. 2000;74: 1215–1219. doi: 10.1016/S0015-0282(00)01618-6 11119753 10.1016/s0015-0282(00)01618-6

[36] Daughtry BL, Chavez SL. Chromosomal instability in mammalian pre-implantation embryos: potential causes, detection methods, and clinical consequences. Cell Tissue Res. 2016;363: 201–225. doi: 10.1007/s00441-015-2305-6 26590822 10.1007/s00441-015-2305-6 PMC5621482

[37] Montag M, Koll B, Holmes P, van der Ven null. Significance of the number of embryonic cells and the state of the zona pellucida for hatching of mouse blastocysts in vitro versus in vivo. Biol Reprod. 2000;62: 1738–1744. 10819778 10.1095/biolreprod62.6.1738

[38] Gordon JW, Dapunt U. A new mouse model for embryos with a hatching deficiency and its use to elucidate the mechanism of blastocyst hatching. Fertil Steril. 1993;59: 1296–1301. 8495780 10.1016/s0015-0282(16)55993-7

[39] Nasr-Esfahani MH, Johnson MH. How does transferrin overcome the in vitro block to development of the mouse preimplantation embryo? J Reprod Fertil. 1992;96: 41–48. 1432973 10.1530/jrf.0.0960041

[40] Duband JL, Thiery JP. Spatio-temporal distribution of the adherens junction-associated molecules vinculin and talin in the early avian embryo. Cell Differ Dev. 1990;30: 55–76. 2112421 10.1016/0922-3371(90)90074-7

[41] Tsuiki A, Preyer J, Hung TT. Effects of fibronectin and its peptide fragment on preimplantation mouse embryo. Am J Obstet Gynecol. 1989;160: 724–728. 2522738 10.1016/s0002-9378(89)80068-7

[42] Fresco VM, Kern CB, Mohammadi M, Twal WO. Fibulin-1 Binds to Fibroblast Growth Factor 8 with High Affinity: EFFECTS ON EMBRYO SURVIVAL. J Biol Chem. 2016;291: 18730–18739. doi: 10.1074/jbc.M115.702761 27402846 10.1074/jbc.M115.702761 PMC5009248

[43] Lahav-Baratz S, Shiloh H, Koifman M, Kraiem Z, Wiener-Megnazi Z, Ishai D, et al Early embryo-endometrial signaling modulates the regulation of matrix metalloproteinase-3. Fertil Steril. 2004;82 Suppl 3: 1029–1035. doi: 10.1016/j.fertnstert.2004.06.026 15474069 10.1016/j.fertnstert.2004.06.026

[44] Reponen P, Leivo I, Sahlberg C, Apte SS, Olsen BR, Thesleff I, et al 92-kDa type IV collagenase and TIMP-3, but not 72-kDa type IV collagenase or TIMP-1 or TIMP-2, are highly expressed during mouse embryo implantation. Dev Dyn. 1995;202: 388–396. doi: 10.1002/aja.1002020408 7626795 10.1002/aja.1002020408

[45] Li R, Yu C, Gao R, Liu X, Lu J, Zhao L, et al Effects of DEHP on endometrial receptivity and embryo implantation in pregnant mice. J Hazard Mater. 2012;241–242: 231–240. doi: 10.1016/j.jhazmat.2012.09.038 23046697 10.1016/j.jhazmat.2012.09.038

[46] Gabrielsen A, Fedder J, Agerholm I. Parameters predicting the implantation rate of thawed IVF/ICSI embryos: a retrospective study. Reprod Biomed Online. 2006;12: 70–76. 16454938 10.1016/s1472-6483(10)60983-2

[47] Zolti M, Ben-Rafael Z, Meirom R, Shemesh M, Bider D, Mashiach S, et al Cytokine involvement in oocytes and early embryos. Fertility and Sterility. 1991;56: 265–272. doi: 10.1016/S0015-0282(16)54483-5 2070856 10.1016/s0015-0282(16)54483-5

[48] Tríbulo P, Siqueira LGB, Oliveira LJ, Scheffler T, Hansen PJ. Identification of potential embryokines in the bovine reproductive tract. J Dairy Sci. 2018;101: 690–704. doi: 10.3168/jds.2017-13221 29128220 10.3168/jds.2017-13221

[49] Robertson SA, Chin PY, Glynn DJ, Thompson JG. Peri-conceptual cytokines—setting the trajectory for embryo implantation, pregnancy and beyond. Am J Reprod Immunol. 2011;66 Suppl 1: 2–10. doi: 10.1111/j.1600-0897.2011.01039.x 21726333 10.1111/j.1600-0897.2011.01039.x

[50] Zhou W, Chu D, Sha W, Fu L, Li Y. Effects of granulocyte-macrophage colony-stimulating factor supplementation in culture medium on embryo quality and pregnancy outcome of women aged over 35 years. J Assist Reprod Genet. 2016;33: 39–47. doi: 10.1007/s10815-015-0627-7 26660059 10.1007/s10815-015-0627-7 PMC4717131

[51] Ziebe S, Loft A, Povlsen BB, Erb K, Agerholm I, Aasted M, et al A randomized clinical trial to evaluate the effect of granulocyte-macrophage colony-stimulating factor (GM-CSF) in embryo culture medium for in vitro fertilization. Fertil Steril. 2013;99: 1600–1609. doi: 10.1016/j.fertnstert.2012.12.043 23380186 10.1016/j.fertnstert.2012.12.043

[52] Barañao RI, Piazza A, Rumi LS, Polak de Fried E. Determination of IL-1 and IL-6 levels in human embryo culture-conditioned media. Am J Reprod Immunol. 1997;37: 191–194. 9083616 10.1111/j.1600-0897.1997.tb00212.x

[53] Goyal P, Brünnert D, Ehrhardt J, Bredow M, Piccenini S, Zygmunt M. Cytokine IL-6 secretion by trophoblasts regulated via sphingosine-1-phosphate receptor 2 involving Rho/Rho-kinase and Rac1 signaling pathways. Mol Hum Reprod. 2013;19: 528–538. doi: 10.1093/molehr/gat023 23538947 10.1093/molehr/gat023

[54] Mehta BN, Chimote NM, Chimote MN, Chimote NN, Nath NM. Follicular fluid insulin like growth factor-1 (FF IGF-1) is a biochemical marker of embryo quality and implantation rates in in vitro fertilization cycles. J Hum Reprod Sci. 2013;6: 140–146. doi: 10.4103/0974-1208.117171 24082656 10.4103/0974-1208.117171 PMC3778604

[55] Han J, Li L, Hu J, Yu L, Zheng Y, Guo J, et al Epidermal growth factor stimulates human trophoblast cell migration through Rho A and Rho C activation. Endocrinology. 2010;151: 1732–1742. doi: 10.1210/en.2009-0845 20150581 10.1210/en.2009-0845

[56] Lim HJ, Dey SK. HB-EGF: a unique mediator of embryo-uterine interactions during implantation. Exp Cell Res. 2009;315: 619–626. doi: 10.1016/j.yexcr.2008.07.025 18708050 10.1016/j.yexcr.2008.07.025 PMC2672567

[57] Artini PG, Valentino V, Monteleone P, Simi G, Parisen-Toldin MR, Cristello F, et al Vascular endothelial growth factor level changes during human embryo development in culture medium. Gynecol Endocrinol. 2008;24: 184–187. doi: 10.1080/09513590801893117 18382903 10.1080/09513590801893117

[58] Cerkiene Z, Eidukaite A, Usoniene A. Immune factors in human embryo culture and their significance. Medicina (Kaunas). 2010;46: 233–239. 20571290

[59] Flamme I, von Reutern M, Drexler HC, Syed-Ali S, Risau W. Overexpression of vascular endothelial growth factor in the avian embryo induces hypervascularization and increased vascular permeability without alterations of embryonic pattern formation. Dev Biol. 1995;171: 399–414. doi: 10.1006/dbio.1995.1291 7556923 10.1006/dbio.1995.1291

[60] Achen MG, Gad JM, Stacker SA, Wilks AF. Placenta growth factor and vascular endothelial growth factor are co-expressed during early embryonic development. Growth Factors. 1997;15: 69–80. 9401819 10.3109/08977199709002113

[61] Komaki M, Numata Y, Morioka C, Honda I, Tooi M, Yokoyama N, et al Exosomes of human placenta-derived mesenchymal stem cells stimulate angiogenesis. Stem Cell Res Ther. 2017;8: 219 doi: 10.1186/s13287-017-0660-9 28974256 10.1186/s13287-017-0660-9 PMC5627451

[62] Hu Y, Rao S-S, Wang Z-X, Cao J, Tan Y-J, Luo J, et al Exosomes from human umbilical cord blood accelerate cutaneous wound healing through miR-21-3p-mediated promotion of angiogenesis and fibroblast function. Theranostics. 2018;8: 169–184. doi: 10.7150/thno.21234 29290800 10.7150/thno.21234 PMC5743467

[63] Lombardo G, Dentelli P, Togliatto G, Rosso A, Gili M, Gallo S, et al Activated Stat5 trafficking Via Endothelial Cell-derived Extracellular Vesicles Controls IL-3 Pro-angiogenic Paracrine Action. Sci Rep. 2016;6: 25689 doi: 10.1038/srep25689 27157262 10.1038/srep25689 PMC4860593

[64] Liu X, Li Q, Niu X, Hu B, Chen S, Song W, et al Exosomes Secreted from Human-Induced Pluripotent Stem Cell-Derived Mesenchymal Stem Cells Prevent Osteonecrosis of the Femoral Head by Promoting Angiogenesis. Int J Biol Sci. 2017;13: 232–244. doi: 10.7150/ijbs.16951 28255275 10.7150/ijbs.16951 PMC5332877

[65] Shabbir A, Cox A, Rodriguez-Menocal L, Salgado M, Van Badiavas E. Mesenchymal Stem Cell Exosomes Induce Proliferation and Migration of Normal and Chronic Wound Fibroblasts, and Enhance Angiogenesis In Vitro. Stem Cells Dev. 2015;24: 1635–1647. doi: 10.1089/scd.2014.0316 25867197 10.1089/scd.2014.0316 PMC4499790

[66] Zhang J, Wang L, Cai L, Cao Y, Duan E. The expression and function of VEGF at embryo implantation “window” in the mouse. ChinSciBull. 2001;46: 409–411. doi: 10.1007/BF03183277

[67] Rappolee DA, Brenner CA, Schultz R, Mark D, Werb Z. Developmental expression of PDGF, TGF-alpha, and TGF-beta genes in preimplantation mouse embryos. Science. 1988;241: 1823–1825. 3175624 10.1126/science.3175624

[68] Artus J, Kang M, Cohen-Tannoudji M, Hadjantonakis A-K. PDGF signaling is required for primitive endoderm cell survival in the inner cell mass of the mouse blastocyst. Stem Cells. 2013;31: 1932–1941. doi: 10.1002/stem.1442 23733391 10.1002/stem.1442 PMC4063320

[69] Betsholtz C, Karlsson L, Lindahl P. Developmental roles of platelet-derived growth factors. Bioessays. 2001;23: 494–507. doi: 10.1002/bies.1069 11385629 10.1002/bies.1069

[70] Hoch RV, Soriano P. Roles of PDGF in animal development. Development. 2003;130: 4769–4784. doi: 10.1242/dev.00721 12952899 10.1242/dev.00721

