# Supplementary figures and images for: Murine embryos exposed to human endometrial MSCs-derived extracellular vesicles exhibit higher VEGF/PDGF AA release, increased blastomere count and hatching rates
Source: PLoS One. 2018 Apr 23;13(4):e0196080. doi: 10.1371/journal.pone.0196080 (PMC5912768; doi:10.1371/journal.pone.0196080)

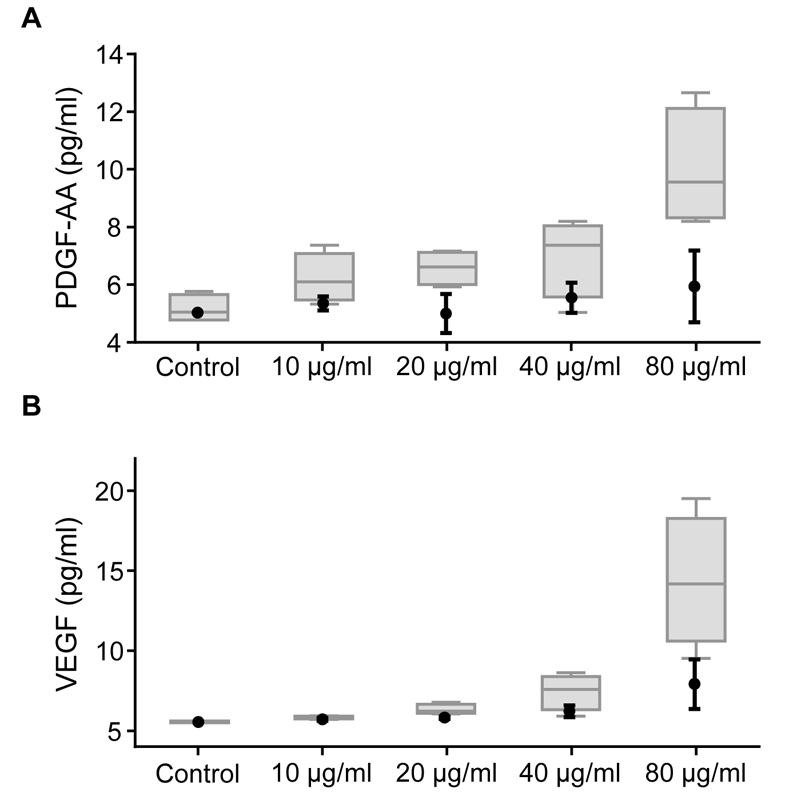

Supplement: S1 Fig — VEGF (A) and PDF-AA (B) concentrations measured by Luminex in experimental samples (box and whiskers diagrams, corresponding to embryos exposed to different concentrations of EV-endMSCs) and negative control samples used as background (plot diagrams, corresponding to culture medium with different concentrations of EV-endMSCs). The means±SD from four independent experiments are shown. (TIF) [file pone.0196080.s001.tif]
